# Development and validation of a predictive nomogram for lower extremity deep vein thrombosis dislodgement in orthopedic patients

**DOI:** 10.3389/fsurg.2023.1148024

**Published:** 2023-03-31

**Authors:** Zongxuan Li, Xiangdong Liu, Liang Li, Pengkai Cao, Guanyu Zhang, Zhipeng Jiao, Fengkai Wang, Qingchun Hao, Yunsong Li, Yanrong Zhang

**Affiliations:** Department of Vascular Surgery, the Third Hospital of Hebei Medical University, Shijiazhuang, China

**Keywords:** lower extremity deep venous thrombosis, pulmonary embolism, risk factors, nomogram, prediction model

## Abstract

**Objective:**

To analyze the risk factors of lower extremity deep venous thrombosis (DVT) detachment in orthopedic patients, and to establish a risk nomogram prediction model.

**Methods:**

The clinical data of 334 patients with orthopedic DVT admitted to the Third Hospital of Hebei Medical University from January 2020 to July 2021 were retrospectively analyzed. General statistics included gender, age, BMI, thrombus detachment, inferior vena cava filter window type, filter implantation time, medical history, trauma history, operation, use of tourniquet, thrombectomy, anesthesia mode, anesthesia grade, operative position, blood loss during operation, blood transfusion, immobilization, use of anticoagulants, thrombus side, thrombus range, D-dimer content before filter implantation and during removal of inferior vena cava filter. Logistic regression was used to perform univariate and multivariate analysis on the possible factors of thrombosis detachment, screen out independent risk factors, establish a risk nomogram prediction model by variables, and internally verify the predictability and accuracy of the model.

**Results:**

Binary logistic regression analysis showed that Short time window filter (OR = 5.401, 95% CI = 2.338–12.478), lower extremity operation (OR = 3.565, 95% CI = 1.553–8.184), use of tourniquet (OR = 3.871, 95% CI = 1.733–8.651), non-strict immobilization (OR = 3.207, 95% CI = 1.387–7.413), non-standardized anticoagulation (OR = 4.406, 95% CI = 1.868–10.390), distal deep vein thrombosis (OR = 2.212, 95% CI = 1.047–4.671) were independent risk factors for lower extremity DVT detachment in orthopedic patients (*P* < 0.05). Based on these six factors, a prediction model for the risk of lower extremity DVT detachment in orthopedic patients was established, and the risk prediction ability of the model was verified. The C-index of the nomogram model was 0.870 (95% CI: 0.822–0.919). The results indicate that the risk nomogram model has good accuracy in predicting the loss of deep venous thrombosis in orthopedic patients.

**Conclusion:**

The nomogram risk prediction model based on six clinical factors, including filter window type, operation condition, tourniquet use, braking condition, anticoagulation condition, and thrombosis range, has good predictive performance.

## 1. Introduction

Deep venous thrombosis (DVT) of the lower extremities is a common clinical disease with an incidence of 1.8%–2.9% (1). Pulmonary embolism (PE) is one of its main complications, which significantly reduces patients’ quality of life and may even endanger their lives. PE is the leading cause of death in patients with lower extremity DVT, and its mortality in developed countries is lower than that of myocardial infarction and tumor (2). The related literature reports that the incidence of PE in orthopedic patients during the perioperative period is about 10%, and the incidence of fatal PE is 0.1%–5.0% (3). For orthopedic patients, a population at high risk of DVT, the mortality rate in the event of a lethal PE is extremely high. The early symptoms of PE are atypical and pose a great challenge to clinical management. Therefore, it is of great significance to predict the risk of lower extremity DVT detachment in orthopedic patients to prevent PE and reduce mortality. Currently, there is a lack of studies on the clinical characteristics of lower extremity DVT detachment in orthopedic patients in China. The paper retrospectively analyzed the clinical data of orthopedic DVT patients who underwent inferior vena cava filter removal in our hospital, discussed the clinical characteristics of thrombus detachment in orthopedic DVT patients, and tried to establish a nomogram prediction model for the risk of lower extremity DVT detachment in orthopedic patients.

## 2. Data and methods

### 2.1. General data

In this study, the clinical data of orthopedic DVT patients recorded in the Donghua Electronic Medical Record System from January 2020 to July 2021 in the Third Hospital of Hebei Medical University were collected according to the pre-established inclusion criteria. Inclusion criteria: (1) Patients with acute lower extremity DVT diagnosed by vascular color Doppler ultrasound of lower extremities; (2) DVT patients who meet the indications of filter implantation (4–6) and underwent inferior vena cava filter implantation in our hospital; (3) Patients with stable thrombus and removal of inferior vena cava filter in our hospital; (4) Patients who underwent orthopedic surgery in our hospital; (5) Those patients who have complete clinical data; (6) Those who can receive telephone follow-up. Exclusion criteria: (1) Patients who did not undergo inferior vena cava filter removal in our hospital; (2) Patients with thrombus involving inferior vena cava; (3) Patients who have incomplete clinical data; (4) Patients with permanent vena cava filter; (5) Patients lost to follow-up. The sample size was estimated using the event per variable (EVP) method, and since the recommended empirical guideline in logistic regression is a sample size of 10–15 times the number of covariates, this study used EPV = 10, involving 26 covariates, so ≥260 patients were required (7). Finally, 334 patients were included in this study. 103 patients were found to have captured thrombus by inferior vena cava filter when receiving inferior vena cava angiography. One patient did not remove the filter because the head end of the filter was embedded in the inferior vena cava wall, and the filter removal rate was 99.7%.

### 2.2. Analysis indicators

Clinical indicators: Totally 26 indicators were collected, including patients’ gender, age, BMI, thrombus detachment, inferior vena cava filter window type, filter implantation time, medical history, trauma history, operation, use of tourniquet, thrombectomy, anesthesia mode, anesthesia grade, operative position, blood loss during operation, blood transfusion, immobilization, use of anticoagulants, thrombus side, thrombus range, D-dimer content before filter implantation and during removal of inferior vena cava filter. The filter window type of inferior vena cava includes short recovery time window filter (the filter is recovered in 2 weeks) and long recovery time window filter (the filter is recovered in more than 2 weeks, and the risk factors of thrombosis and progression are reduced or eliminated, so it should be recovered as soon as possible, in principle, no more than 3 months. The recovery time can be appropriately extended if necessary). The short-window spindle filters included in this study include OptEase, Aegisy, and Illicium, and the long-window cone filters include Denali and Option. Strict immobilization means that the affected limb is not allowed to move and keep a fixed position during treatment, while non-strict immobilization means that patients can adequately move their lower extremities and walk around during treatment. Standardized anticoagulation refers to the sufficient dose (low molecular weight heparin: 100 U per kilogram of body weight, one subcutaneous injection every 12 h; Rivaroxaban: 15 mg 2/day in the first 3 weeks, maintenance period: 20 mg 1/day), sufficient anticoagulation during treatment course (anticoagulation treatment for DVT patients with definite inducement for 3 months, anticoagulation for DVT patients without definite incentive for at least 3 months).

### 2.3. Statistical method

All the data in this study were analyzed by SPSS 26.0. The chi-square test or Fisher exact test was used for counting data. All data were randomly divided into a training set and validation set by R software (4.2.0) at a sample size of 7:3. Univariate and multivariate logistic regression analysis was used to analyze the risk factors of lower extremity DVT detachment in orthopedic patients in the training set. The independent variable *P* < 0.2 in univariate analysis was included in the multivariate regression analysis. The logistic regression model with the least amount of information in the Akaike information criterion (AIC) was selected as the final prediction model, and the visual output was carried out by using R software through a nomogram. The calibration curve was plotted to compare actual risk with predicted risk; the decision curve analysis (DCA) and clinical impact curve (CIC) were plotted to evaluate the clinical application value of the nomogram by calculating the net benefit under different threshold probabilities. The receiver operating character (ROC) curve was plotted to assess discrimination and calibration of the model by the area under curve (AUC).

## 3. Results

### 3.1. Comparison of general data between the training set and validation set

After comparison, the difference was not statistically significant (*P* > 0.05) between the two groups in patients’ gender, age, BMI, thrombus detachment, inferior vena cava filter window type, filter implantation time, medical history, trauma history, operation, use of tourniquet, thrombectomy, anesthesia mode, anesthesia grade, operative position, blood loss during operation, blood transfusion, immobilization, use of anticoagulants, thrombus side, thrombus range, D-dimer content before filter implantation and during removal of inferior vena cava filter, as shown in Table 1.

**Table 1:** Comparison of general data between training set and validation set.

Factor	Modeling group (*n* = 235)	Test group (*n* = 99)	Statistical value	*P* value
Thrombus detachment [case (%)]	73 (31.1)	30 (30.3)	0.019	0.891
Male [case (%)]	142 (60.4)	63 (63.6)	0.303	0.582
Age [case (%)]			0.411	0.814
Age ≤ 44	60 (25.5)	24 (24.2)		
45 ≤ age ≤ 59	70 (29.8)	33 (33.3)		
Age ≥ 60	105 (44.7)	42 (42.4)		
BMI [case (%)]			2.063^a^	0.570
<18.5	8 (3.4)	2 (2.0)		
18.5 ≤ BMI < 24	78 (33.2)	33 (33.3)		
24 ≤ BMI <28	94 (40.0)	46 (46.5)		
≥28	55 (23.4)	18 (18.2)		
Short time window filter [case (%)]	98 (41.7)	37 (37.4)	0.542	0.462
Filter implantation > 14 days [case (%)]	182 (77.4)	80 (80.8)	0.465	0.495
Hypertension [case (%)]	64 (27.2)	33 (33.3)	1.257	0.262
Coronary heart disease [case (%)]	18 (7.7)	12 (12.1)	1.696	0.193
Diabetes [case (%)]	27 (11.5)	12 (12.1)	0.027	0.870
Trauma [case (%)]	203 (86.4)	84 (84.8)	0.136	0.713
Lower extremity surgery [case (%)]	138 (58.7)	54 (54.5)	0.497	0.481
Multiple operations [case (%)]	38 (16.2)	18 (18.2)	0.202	0.653
Thrombectomy [case (%)]	12 (5.1)	2 (2.0)	0.973^a^	0.324
Time between injury and operation ≤14 days [case (%)]	190 (80.9)	82 (82.8)	0.180	0.671
Tourniquet [case (%)]	67 (28.5)	27 (27.3)	0.053	0.818
Operation duration < 2 h [case (%)]	78 (33.2)	29 (29.3)	0.486	0.486
General anesthesia [case (%)]	200 (85.1)	82 (82.8)	0.275	0.600
ASA Grade (I, II) [case (%)]	181 (77.0)	76 (76.8)	0.003	0.960
Operative position (supine) [case (%)]	184 (78.3)	82 (82.8)	0.882	0.348
Blood loss during operation [case (%)]			4.822	0.090
None	17 (7.2)	10 (10.1)		
0<blood loss ≤ 500ml	149 (63.4)	50 (50.5)		
≥500ml	69 (29.4)	39 (39.4)		
Intraoperative blood transfusion [case (%)]	68 (28.9)	35 (35.4)	1.345	0.246
Strict immobilization [case (%)]	175 (74.5)	70 (70.7)	0.504	0.478
Standardized anticoagulation [case (%)]	159 (67.7)	67 (67.7)	0.000	0.998
Distal DVT [case (%)]	133 (56.6)	50 (50.5)	1.043	0.307
Thrombus side [case (%)]			0.141	0.932
Left	99 (42.1)	42 (42.4)		
Right	82 (34.9)	36 (36.4)		
Both	54 (23.0)	21 (21.2)		
D-dimer increase when filter was implanted [case (%)]	228 (97.0)	98 (99.0)	0.466^a^	0.495
D-dimer increase when filter was taken out [case (%)]	162 (68.9)	70 (70.7)	0.103	0.748

^a^ Denotes corrected chi-square test results.

### 3.2. Clinical indicator analysis

The results of univariate analysis of the training set (see Table 2) show that: the related factors (*P* < 0.2) of lower extremity DVT detachment in orthopedic patients were the filter window type, operation condition, multiple operations, thrombectomy, the time between injury and operation, use of tourniquet, strict immobilization, standardized anticoagulation, thrombus range and D-dimer increase when the filter was taken out. Binary logistic regression analysis showed that the independent risk factors (*P* < 0.05) of lower extremity DVT detachment in orthopedic patients were short time window filter, lower extremity surgery, use of tourniquet, non-strict immobilization, non-standardized anticoagulation, and distal DVT, as shown in Table 3.

**Table 2:** Univariate analysis of risk factors of lower extremity DVT detachment in orthopedic patients in training set.

Clinical factors	Thrombus detachment group (*n* = 73)	Non-thrombus detachment group (*n* = 162)	Wald value	OR value (95% CI)	*P* value
Gender (Female^a^/Male)	27/46	66/96	0.296	1.171 (0.663–2.070)	0.586
**Age (case)**					
Age ≤ 44^a^	17	43			
45 ≤ age ≤ 59	24	46	0.529	1.320 (0.625–2.787)	0.467
Age ≥ 60	32	73	0.084	1.109 (0.551–2.230)	0.772
**BMI (case)**					
<18.5^a^	3	5			
18.5 ≤ BMI < 24	27	51	0.027	0.882 (0.196–3.976)	0.871
24 ≤ BMI < 28	27	67	0.271	0.672 (0.150–3.008)	0.603
≥28	16	39	0.233	0.684 (0.146–3.206)	0.630
Filter window type (long time window^a^/short time window)	27/46	110/52	18.871	3.604 (2.021–6.427)	0.000
Days of filter implantation (≤14 days^a^/>14 days)	19/54	34/128	0.729	0.755 (0.396–1.439)	0.393
Hypertension (No^a^/Yes)	53/20	118/44	0.001	1.012 (0.544–1.881)	0.970
Coronary heart disease (No^a^/Yes)	68/5	149/13	0.098	0.843 (0.289–2.458)	0.754
Diabetes (No^a^/Yes)	62/11	146/16	1.316	1.619 (0.711–3.687)	0.251
Trauma (No^a^/Yes)	12/61	20/142	0.712	0.716 (0.330–1.556)	0.399
Operation condition (non-lower extremity operation^a^/lower extremity operation)	14/59	83/79	19.576	4.428 (2.291–8.559)	0.000
Multiple operations (No^a^/Yes)	68/5	132/30	2.075	0.542 (0.235–1.248)	0.150
Thrombectomy (No^a^/Yes)	67/6	156/6	2.014	2.328 (0.725–7.481)	0.156
Time between injury and operation (≤14 days^a^/>14 days)	64/9	126/36	3.093	0.492 (0.223–1.084)	0.079
Tourniquet (No^a^/Yes)	35/38	133/29	26.597	4.979 (2.705–9.165)	0.000
Operation duration (≥2 h^a^/<2 h)	49/24	108/54	0.005	0.98 (0.544–1.763)	0.945
Anesthesia mode (general anesthesia^a^/non-general anesthesia)	64/9	136/26	0.547	0.736 (0.326–1.660)	0.460
Anesthesia grade [ASA (I, II)^a^/ASA (III, IV)]	60/13	121/41	1.584	0.639 (0.319–1.283)	0.208
Operative position (supine^a^/non-supine)	59/14	125/37	0.396	0.802 (0.403–1.596)	0.529
**Blood loss during operation (case)**					
0^a^	6	11			
<500ml	49	100	0.040	0.898 (0.314–2.572)	0.842
≥500ml	18	51	0.570	0.647 (0.209–2.004)	0.450
Intraoperative blood transfusion (No^a^/Yes)	52/21	115/47	0.001	0.988 (0.537–1.818)	0.969
Strict immobilization (No^a^/Yes)	40/33	135/27	20.132	4.125 (2.221–7.661)	0.000
Standardized anticoagulation (No^a^/Yes)	42/31	117/45	4.893	1.919 (1.077–3.419)	0.027
Thrombus range (proximal DVT^a^/distal DVT)	27/46	75/87	1.767	1.469 (0.833–2.589)	0.184
**Thrombus side (case)**					
Left^a^	29	70			
Right	25	57	0.031	1.059 (0.559–2.006)	0.861
Both	19	35	0.562	1.310 (0.646–2.656)	0.453
D-dimer increase when filter was implanted (No^a^/Yes)	3/70	4/158	0.459	0.591 (0.129–2.709)	0.498
D-dimer increase when filter was taken out (No^a^/Yes)	13/60	60/102	8.309	2.715 (1.377–5.354)	0.004

^a^ Represents the control group.

**Table 3:** Multivariate analysis of risk factors of lower extremity DVT detachment in orthopedic patients in training set.

Factor	B	SE	Wald-*χ*	*P* value	OR (95% CI)
Short recovery time window filter	1.687	0.427	15.583	0.000	5.401 (2.338–12.478)
Lower extremity operation	1.271	0.424	8.986	0.003	3.565 (1.553–8.184)
Use of tourniquet	1.354	0.410	10.887	0.001	3.871 (1.733–8.651)
Non-strict immobilization	1.165	0.428	7.429	0.006	3.207 (1.387–7.413)
Non-standardized anticoagulation	1.483	0.438	11.475	0.001	4.406 (1.868–10.390)
Distal DVT	0.794	0.381	4.332	0.037	2.212 (1.047–4.671)

### 3.3. Drawing and validation of a nomogram

**Figure 1:**
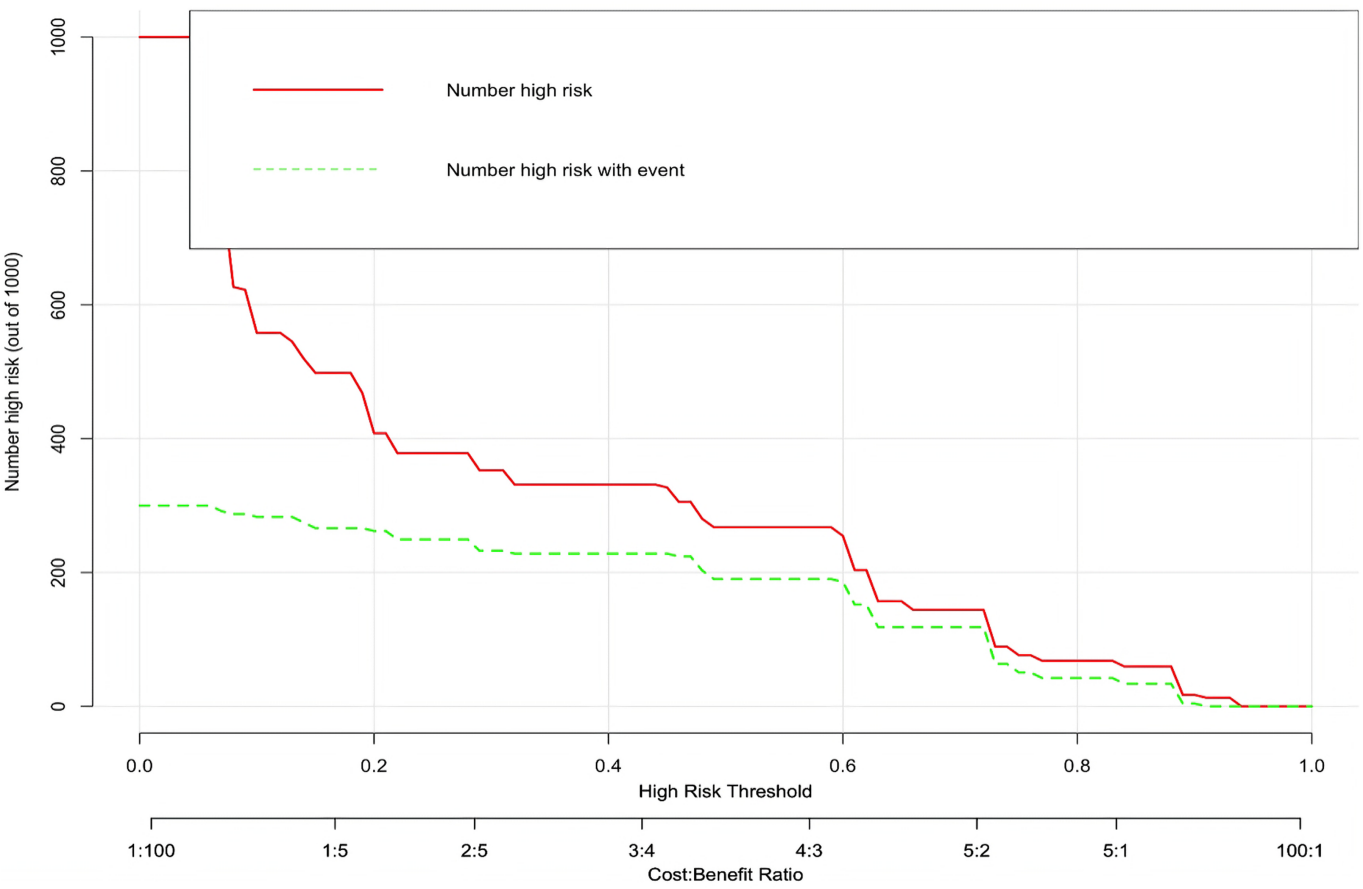
Establishment of nomogram risk model for predicting lower extremity DVT detachment in orthopedic patients.

**Figure 2:**
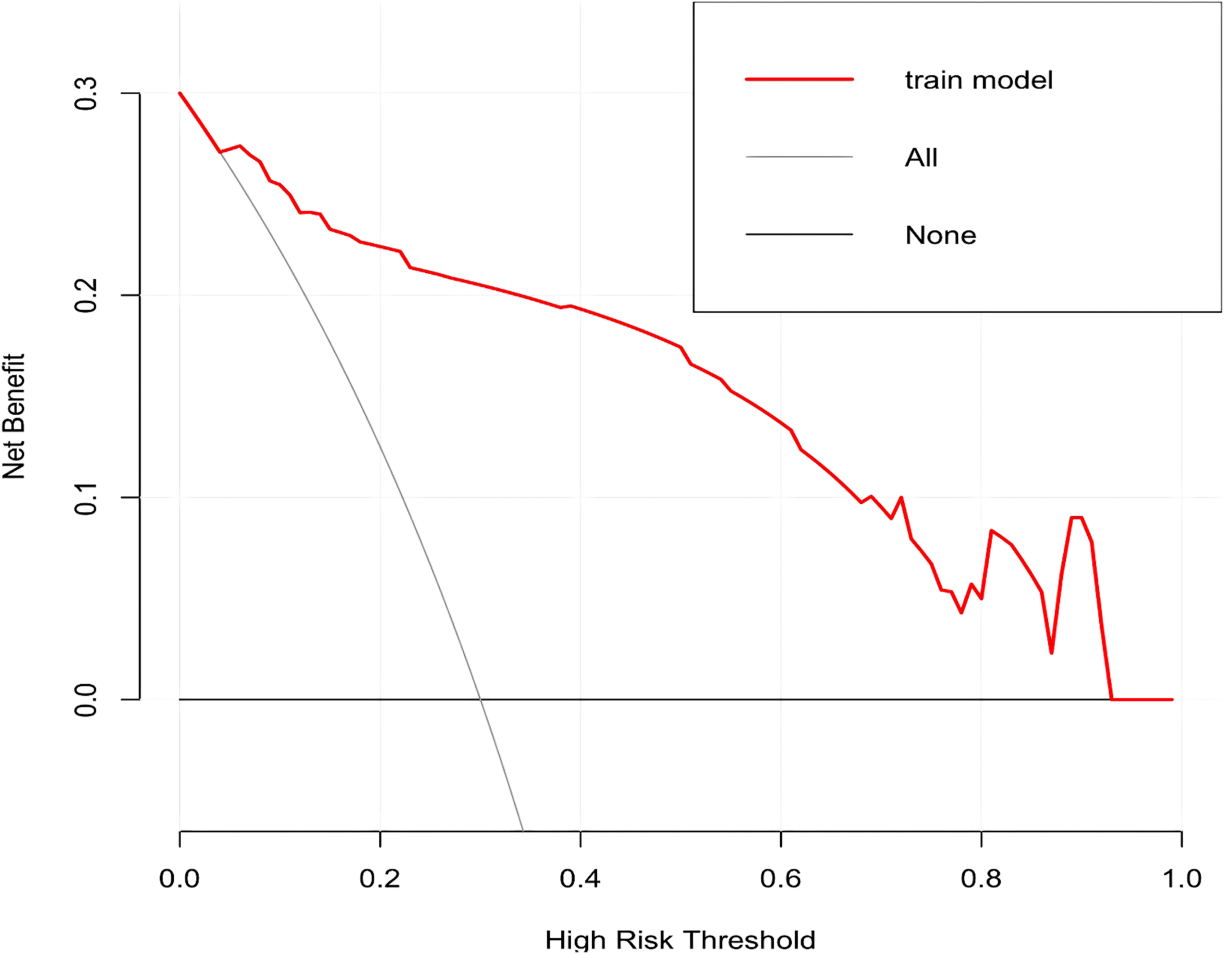
Calibration curve of nomogram model for predicting the risk of lower extremity DVT detachment in orthopedic patients.

**Figure 3:**
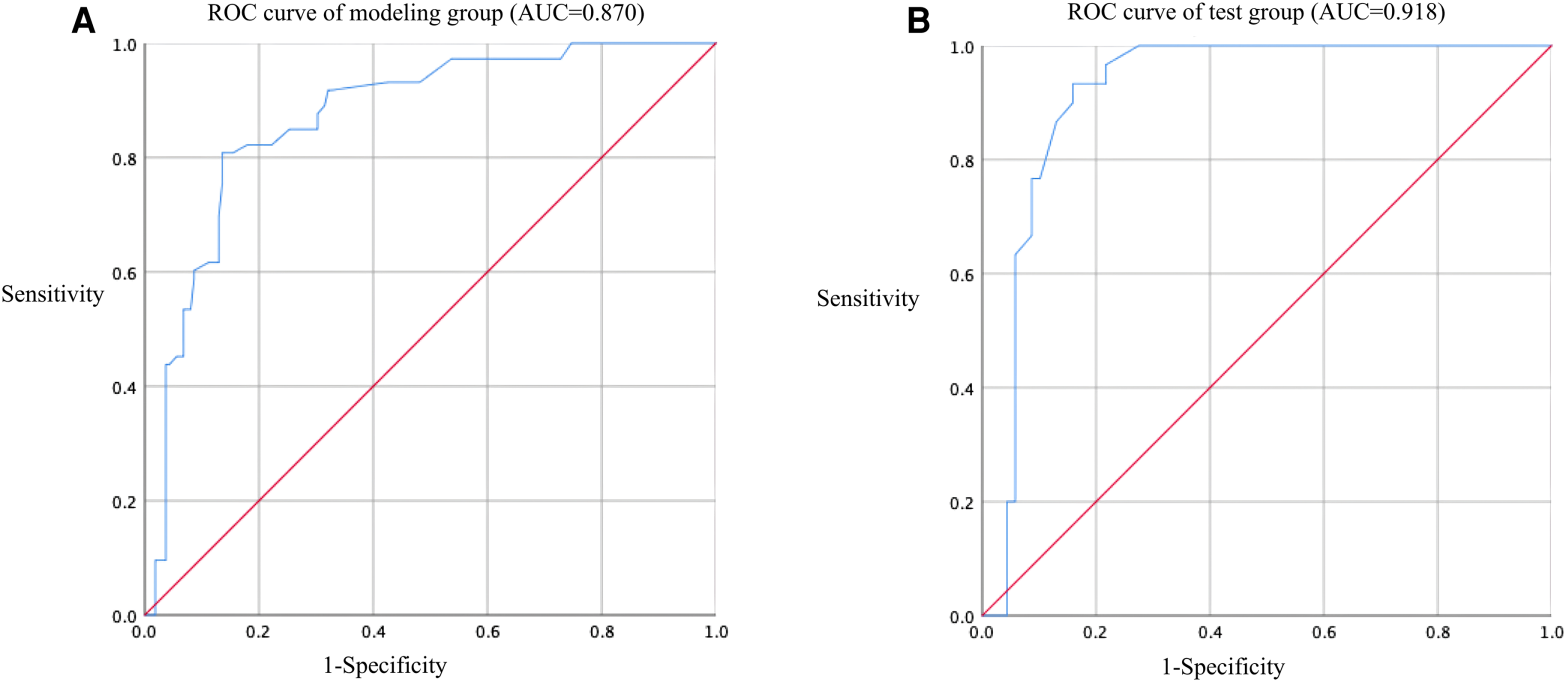
ROC curve of nomogram model for predicting the risk of lower extremity DVT detachment in orthopedic patients. (**A**) ROC curve of modeling group (AUC = 0.870); (**B**) ROC curve of test group (AUC = 0.918).

**Figure 4:**
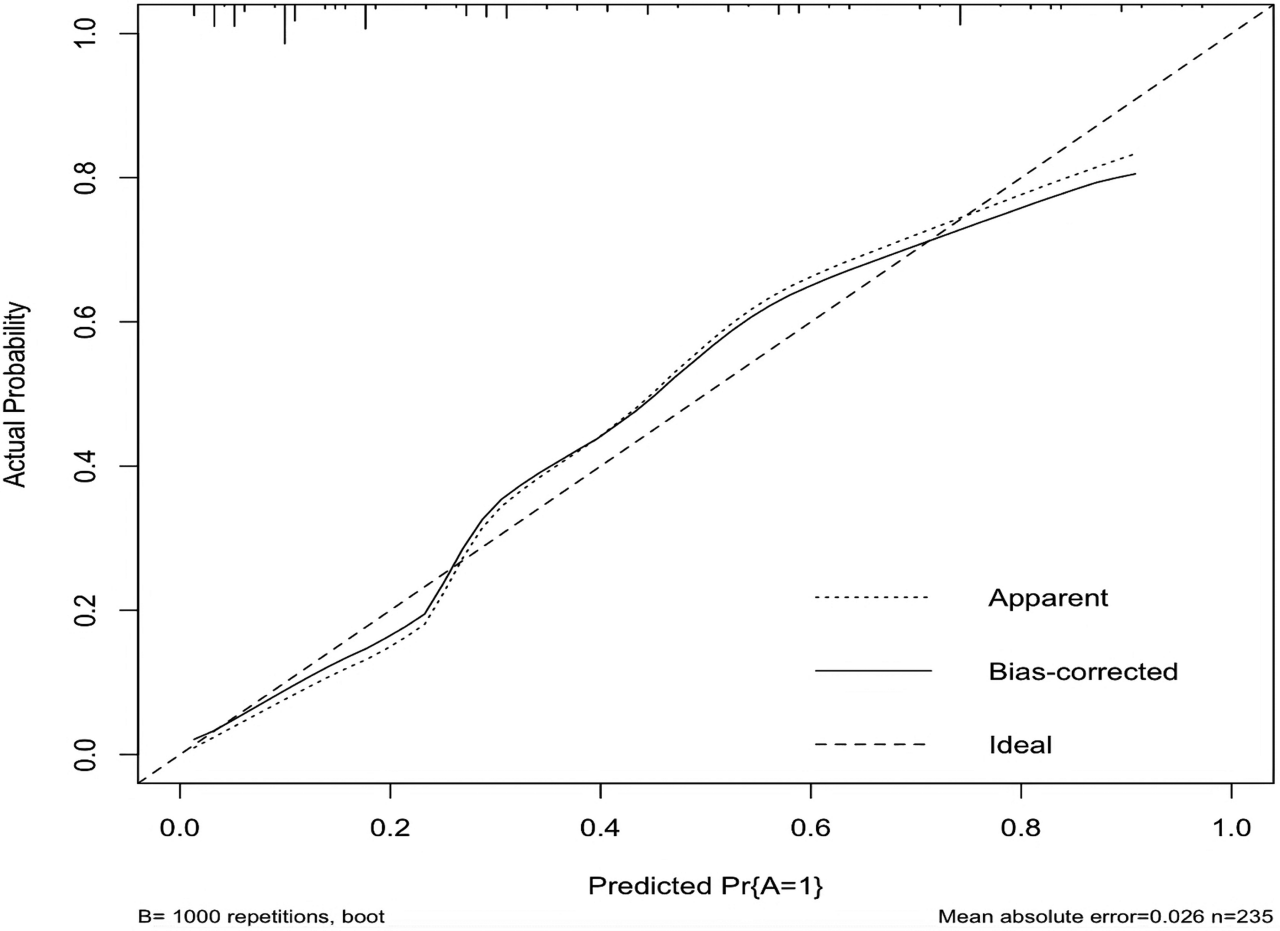
Clinical decision curve of nomogram model.

**Figure 5:**
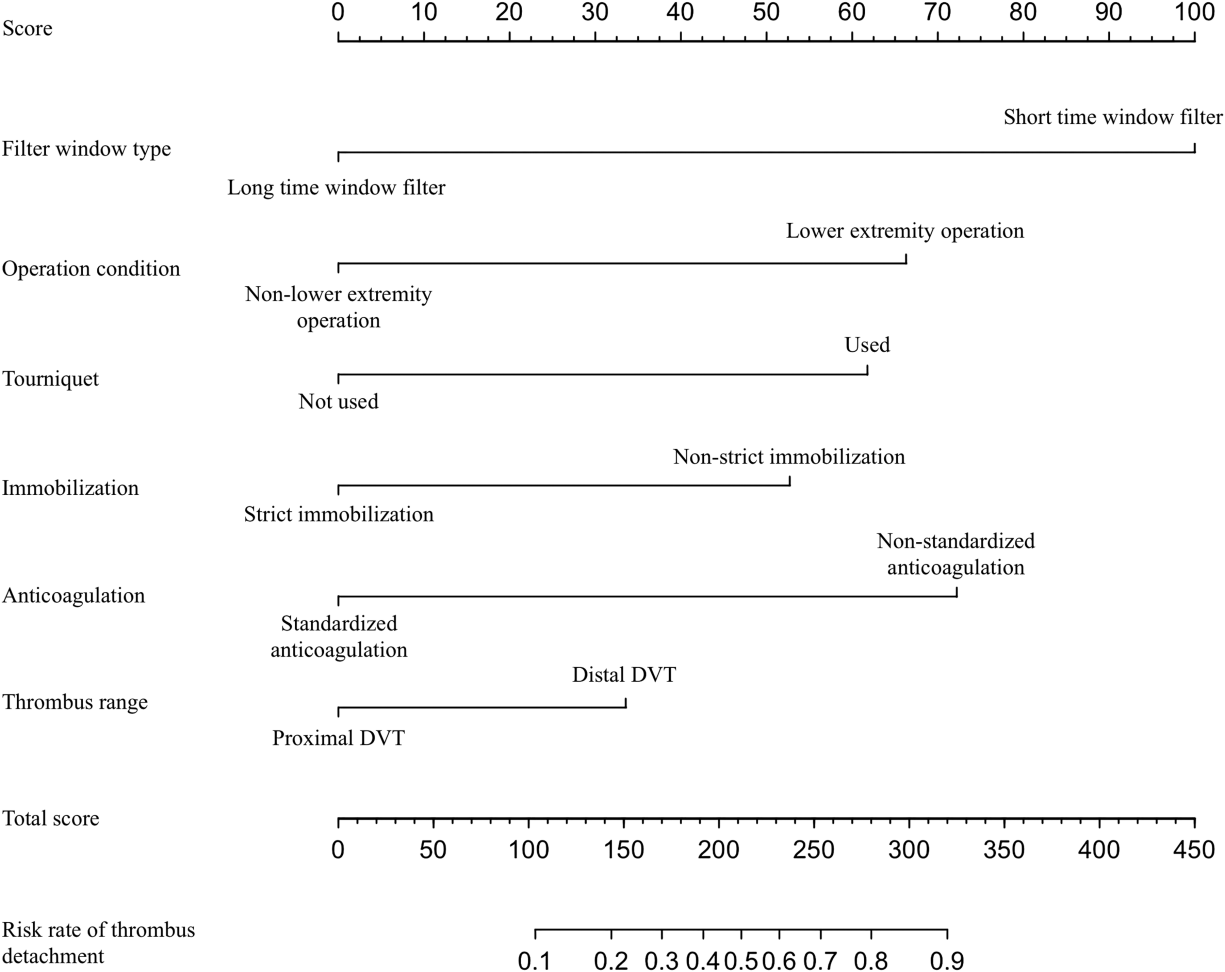
Clinical impact curve of nomogram model.

In this study, based on the comparison of univariate and multivariate logistics, six independent risk factors were obtained to establish a nomogram prediction risk model for predicting lower extremity DVT detachment in orthopedic patients (Figure 1). The model was internally validated by the parallel Bootstrap method (after repeated sampling of the included data 1,000 times), and the calibration curve was close to the ideal curve (Figure 2), which showed that lower extremity DVT detachment rate in orthopedic patients predicted by this nomogram was highly consistent with the actual detachment rate. The ROC curve drawn by the nomogram (Figure 3) shows that the training set AUC is 0.870 (95% CI: 0.822–0.919, Sensitivity: 80.8%, Specificity: 86.4%), and the test assigned AUC is 0.918 (95% CI: 0.861–0.975, Sensitivity: 93.3%, Specificity: 84.1%), indicating that the prediction risk model of the nomogram has a reasonable degree of discrimination and accuracy for the high-risk population of thrombus detachment. The clinical decision curve shows that the risk prediction model of this nomogram provides a more significant net benefit than the strategies of “intervention for all” and “no intervention for all,” indicating that this model has a higher clinical application value (see Figure 4). The clinical impact curve drawn by the nomogram represents the number of high-risk people judged by the model and the actual number of faithful positive people under different threshold probabilities, which can more intuitively reflect that the prediction model has a higher clinical net benefit (see Figure 5).

## 4. Discussion

DVT is a common disease in inpatients, but the risk of DVT in orthopedic patients is more elevated, mainly due to the following reasons: injury of vascular intima; slow venous blood flow caused by long-term bed rest or affected limb immobilization; fibrinolytic inhibition caused by trauma or surgery; activating coagulation process, leading to a transient hypercoagulable state, thus causing local blood coagulation and DVT (8). PE caused by lower extremity DVT detachment is a serious complication of DVT. Once fatal PE occurs, mortality is extremely high. In clinical practice, because most of the early symptoms of PE are not evident and atypical, it brings difficulties to clinical diagnosis and treatment. Therefore, starting with the risk factors of thrombus detachment, analyzing clinical data, integrating and constructing risk prediction models, and identifying the high-risk population of thrombus detachment will contribute to the early diagnosis and treatment of PE.

This study found that the short recovery time window filter (shaped as “spindle,” also known as spindle filter) is easier to capture thrombus than the long recovery time window filter (shaped as “cone,” also known as cone filter). The reasons may be as follows: First, the shapes of filters are different. The spindle-shaped filter rod is in “rod contact” with the inferior vena cava, while the cone-shaped filter rod is in “point contact” with the inferior vena cava, which has a larger contact area and is easier to change the hemodynamics of the inferior vena cava (9); Second, the anticoagulation duration is different. The cone-shaped filter generally has a longer take-out time window than the spindle filter. During the protection of the filter, the anticoagulation time is longer, which is more beneficial to thrombolysis, i.e., the thrombus is captured within 2 weeks after the filter is implanted. With the extension of anticoagulation time, the thrombus in the filter is gradually dissolved with the recovery of the fibrinolytic system of the human body, and it may become progressively smaller or disappear.

This study found that the lower extremity DVT detachment in orthopedic patients was related to the application of tourniquets during operation. In lower extremity surgery, orthopedic surgeons often reduce intraoperative bleeding by “dispersing blood” before operation and applying tourniquets, so as to obtain a clear surgical field of vision and facilitate intraoperative operation. Darmanis et al. reported two cases of pulmonary embolism immediately after the tourniquet was applied to “dispersing blood” in patients undergoing lower extremity surgery, and the final rescue failed (10). The author considers that either “dispersing blood” or applying tourniquets may change the hemodynamics in blood vessels of lower extremities, promote the change of coagulation function, and have an impact on the formation and prognosis of lower extremity DVT. The reasons are as follows: (1) “dispersing blood” and applying tourniquets will keep the lower extremity in a long-term venous congestion state, lead to tissue ischemia and hypoxia, aggravate the hypercoagulable state of blood, and promote acute lower extremity DVT during operation (11); (2) Preoperative “dispersing blood” can make the thrombus move from the distal end to the proximal end, and be intercepted by the tourniquet at the thigh root. With the continuous displacement and detachment of the thrombus during operation, small thrombi gradually converge into the large thrombus, and the old thrombus slowly grows into a new thrombus (10); (3) When the tourniquet is loosened at the end of the operation, due to the recovery of venous blood flow, a large number of thrombi intercepted by the tourniquet detach, resulting in pulmonary embolism or fatal pulmonary embolism. Therefore, if DVT is shown by color doppler ultrasound before the operation, the operation time can be delayed for patients with lower extremity fractures. The tourniquet can be used to complete the operation based on adequate anticoagulation, which can reduce the risk of pulmonary embolism caused by the progression of lower extremity DVT or thrombus detachment during and after the operation.

In this study, multivariate results show that lower extremity surgery is an independent risk factor for lower extremity DVT detachment in orthopedic patients; that is, lower extremity surgery is more likely to cause thrombosis detachment than non-lower extremity surgery for patients, as with the study report of Godzik et al. (12). In lower extremity fracture surgery, surgical instruments such as retractors will inevitably be used for visual field exposure. Intermittent squeezing and pulling of retractor and operation may cause compression and even damage to muscles and blood vessels of lower extremities, which significantly increases the risk of DVT occurrence during operation, not only making fresh thrombus easily detach to the proximal end, but also making old or stable thrombi move under the action of external force.

Multivariate analysis showed that non-strict immobilization and non-standardized anticoagulation were independent risk factors for thrombus detachment. Strict immobilization of lower extremities may prevent large thrombus from separating from the thrombus wall before thrombus organization. In comparison, patients with free movement of lower extremities have a higher risk of thrombus detachment from the blood vessel wall. As the primary treatment of thrombosis, standardized anticoagulation can delay the progress of thrombosis and rely on the fibrinolytic system in patients to dissolve thrombosis. During the treatment of DVT, if standardized anticoagulation is not given and thrombosis is in the acute stage, with the reflux of venous blood, some thrombi will detach from the lower extremities and cause PE. Although many early clinical studies reported that under standardized anticoagulation, there was no statistical difference in PE incidence between immobilized patients and active patients (13–15), in the current clinical practice, patients are not prematurely encouraged to move on the ground during the treatment of DVT. The possible reasons are as follows: During the treatment of acute DVT, it often gets into a dilemma: clinicians often need to consider the consequences of thrombus progression caused by long-term bed-rest immobilization, thrombus detachment and pulmonary embolism caused by ground activities, high bleeding tendency accompanied by anticoagulation and thrombus spreading and division caused by non-standardized anticoagulation. They need to consider not only the patient's life but also their financial condition and treatment compliance. Therefore, the author thinks that strict immobilization under standardized anticoagulant therapy, that is, moving around only after the thrombus is stable and organized, may reduce complications.

This study found that distal DVT has a higher risk of detachment than proximal DVT, which is contrary to most current study conclusions (16, 17), possibly because clinicians pay less attention to distal DVT. Konstantinides et al. observed that distal DVT was not easy to detach and did not directly lead to pulmonary embolism, which may be why distal DVT is neglected (18). In addition, most of the orthopedic patients included in this study were complicated with trauma. In order to avoid bleeding complications, distal DVT may not be fully treated. It is reported in the literature that the incidence of pulmonary embolism caused by intermuscular venous thrombosis in the lower legs is 7%–50% (19). Therefore, we need to pay attention to distal DVT and give appropriate treatment to avoid its spreading, as it may even lead to fatal pulmonary embolism.

To summarize, filter window type, operation condition, use of tourniquet, non-strict immobilization, non-standardized anticoagulation, and thrombus range are independent risk factors for lower extremity DVT detachment in orthopedic patients, and the nomogram risk prediction model established has good prediction performance and high clinical value. This study inevitably has its limitations, Firstly, the sample size is still not very adequate, and even if each independent variable EPV is taken as 10 as the sample size for the dichotomous logistic regression, it will still underestimate the reasonable sample size level, which may lead to instability of the multi-factor logistic regression results; Second, the types of short- and long-window filters were not completely uniform, and the variability in the structure of the same time-window filter itself may lead to differences in their ability to capture thrombus. In addition, due to the inadequate sample size of this study, the nomogram has only been internally validated, and its extrapolation is relatively insufficient, and in the future large sample and multi-center studies are needed to provide more theoretical support for this prediction model.

## 5. Conclusion

Filter window type, surgical situation, tourniquet use, braking situation, anticoagulation situation, and thrombus extent are independent risk factors for lower limb DVT dislodgement in orthopedic patients, and its nomogram model can individually predict the risk of lower limb DVT dislodgement in orthopedic patients, which has high clinical value.

## Data Availability

The original contributions presented in the study are included in the article/supplementary material, further inquiries can be directed to the corresponding author/s.
